# A Comparative Analysis of the Microbiome of Kiwifruit at Harvest Under Open-Field and Rain-Shelter Cultivation Systems

**DOI:** 10.3389/fmicb.2021.757719

**Published:** 2021-09-30

**Authors:** Yuan Sui, Qianhua Zhao, Zhenshuo Wang, Jia Liu, Mingguo Jiang, Junyang Yue, Jianbin Lan, Jing Liu, Qinhong Liao, Qi Wang, Qiya Yang, Hongyin Zhang

**Affiliations:** ^1^Chongqing Key Laboratory of Economic Plant Biotechnology, College of Landscape Architecture and Life Science/Institute of Special Plants, Chongqing University of Arts and Sciences, Yongchuan, Chongqing, China; ^2^School of Food and Biological Engineering, Jiangsu University, Zhenjiang, China; ^3^Department of Plant Pathology, MOA Key Lab of Pest Monitoring and Green Management, College of Plant Protection, China Agricultural University, Beijing, China; ^4^Engineering Research Center of Plant Growth Regulators/Crop Chemical Control Research Center, Department of Agronomy, College of Agronomy and Biotechnology, China Agricultural University, Beijing, China; ^5^Guangxi Key Laboratory for Polysaccharide Materials and Modifications, School of Marine Sciences and Biotechnology, Guangxi University for Nationalities, Nanning, China; ^6^College of Horticulture, Anhui Agricultural University, Hefei, China

**Keywords:** *Actinidia chinensis*, cultivation mode, disease incidence, microbial community, fruit quality

## Abstract

The composition of microbial communities can directly affect fruit quality, health status, and storability. The present study characterized the epiphytes and endophytes of “Hongyang” and “Cuiyu” kiwifruit at harvest under grown under open-field (OF) and rain-shelter (RS) cultivation systems. Disease incidence in kiwifruit was significantly lower (*p* < 0.05) under the RS system than it was under the OF system. High-throughput sequencing [16S V3-V4 ribosomal region and the fungal internal transcribed spacer (ITS2)] was conducted to compare the composition of the epiphytic and endophytic microbial community of kiwifruit under the two cultivation systems. Results indicated that the abundance of Actinobacteria, Bacteroidetes, Enterobacteriales, Acetobacterales, *Sphingomonas*, *Pseudomonas*, and *Sphingobacterium* was higher under the RS system, relative to the OF system, while the abundance of Capnodiales, Hypocreales, *Vishniacozyma*, and *Plectosphaerella* was also higher under the RS system. Some of these bacterial and fungal taxa have been reported to as act as biocontrol agents and reduce disease incidence. Notably, the α-diversity of the epiphytic bacterial and fungal communities on kiwifruit was higher under RS cultivation. In summary, RS cultivation reduced natural disease incidence in kiwifruit, which may be partially attributed to differences in the structure and composition of the microbial community present in and on kiwifruit.

## Introduction

Kiwifruit, known as the “king of fruits,” has a high nutraceutical value. Kiwifruit is rich in bioactive compounds with antioxidant properties and is a major source of vitamin C (Richardson et al., 2018). Kiwifruit fruits, however, are susceptible to various plant pathogens, including *Botrytis cinerea* and *Alternaria alternata* (Cheng et al., 2019), *Pseudomonas syringae* (Fujikawa et al., 2020), and Actinidia viruses (Zhao L. et al., 2019), which cause a variety of diseases during growth (Ippolito and Nigro, 2000). In order to reduce disease incidence in plants and increase their economic value, rain-shelters (RS) have been used in the cultivation of several different fruits, including pear (Zhang et al., 2020), grape (Meng et al., 2012), and cherry (Tian et al., 2019). Collectively, studies have demonstrated that RS cultivation prevents excessive rainfall and hail damage to flowers and fruits and reduces disease occurrence, thus providing significant protection (Tian et al., 2019). Meng et al. (2012) reported that RS cultivation significantly reduced the incidence of grape diseases, delayed fruit ripening, and promoted the accumulation of quercetin-3-O-glucose-7-O-rhamnoside in grape skin peels during grape ripening. Claire et al. (2018) also found that RS cultivation reduced the level of fruit rot in strawberries. In the present study, RS cultivation was used to reduce the occurrence of kiwifruit natural disease and to explore the impact of RS cultivation on the microbiome of kiwifruit.

The plant microbiome plays an integral role in plant health and productivity, and has been the subject of a great deal of research in recent years (Turner et al., 2013). While the microbiome of many vegetable and field crops has been explored and characterized, much less attention has been paid to fruit crops (Busby et al., 2017). The epiphytic microbial community has a positive influence on plant health, physiology, and environmental adaptability (Kim et al., 2011; Vorholt, 2012; Dees et al., 2015). Biological control agents (BCAs) represent an environmentally friendly alternative to the use of chemical pesticides for disease control (Spadaro and Droby, 2016). Several epiphytic bacterial species isolated from the phyllosphere have been reported to be strong competitors against plant pathogens, thus acting as BCAs (Enya et al., 2007; Sartori et al., 2017). They can secrete antimicrobial substances (Insuk et al., 2020; López et al., 2020), compete for nutrients and space in a limited space, or interfere with the signaling systems of pathogens (Volksch and May, 2001). Dong et al. (2021) studied the epiphytic and endophytic fungal communities of tomato plant roots, leaves, fruits, and seeds. Some endophytic fungi, including yeast, have been proved to be inhibitors of post-harvest diseases. Endophytic yeast fungi isolated from apple were also found to have an inhibitory effect on *Monilinia fructigena*, the causal agent of soft-rot in apple (Madbouly et al., 2020). The endophytic bacteria present in tomato plants has been reported to play an important role in fostering plant growth and health (López et al., 2020). These reports indicate that microbial community composition is linked to levels of plant disease. Mamphogoro et al. (2020) reported a greater abundance of potential pathogenic bacteria on the fruit surface of immature (green) and mature (red) pepper planted under open field (OF) conditions, compared to peppers planted in a hydroponic system. Their data indicated that the microbial community of plants is affected by cultivation methods. Understanding the distribution and composition of the epiphytic and endophytic microbial community of plants may provide important information on how the RS cultivation of kiwifruit reduces the occurrence of natural disease.

In the present study, high-throughput 16S and fungal ITS amplicon sequencing was used to characterize the microbial community of kiwifruit peel and pulp tissues from fruit that was harvested from OF and RS cultivation systems. The objectives were to (i) profile the bacterial and fungal microbial taxa present in and on kiwifruit, and (ii) determine whether cultivar and the two different cultivation systems (RS and OF) influenced the structure and composition of the resident microbiota in a manner that reduced disease incidence.

## Materials and Methods

### Experimental Site Description

The experimental site in this study was located at Huanggua Mountain, Yongchuan District, Chongqing City, China (29°25′47″N, 105°84′96″E). Two kiwifruit (*Actinidia chinensis*) cultivars, “Hongyang” and “Cuiyu,” were grown under OF and RS conditions, with the latter being provided with the use of 0.12-mm anti-fog film (6-m height and 6-m width). A total of 20 plants were covered with polyethylene (PE) film from flowering (April) to fruit harvest (September) while another 20 were left uncovered. Each group (OF and RS) of plants contained 10 plants of “Hongyang” and 10 plants of “Cuiyu.” No pesticides were applied to either group of plants over the course of the present study.

### Disease Incidence and Sample Preparation

Incidence data was collected in late Septembers (the harvest time of kiwifruit) in the 2 years of 2018 and 2019. Six plants were randomly selected from a group of 10 plants. Disease incidence was assessed on three replicate groups of fruit, with each treatment group comprising 120 fruits. Natural disease incidence was calculated as a percentage [number of fruits with visible disease symptoms (Fruits with visible spots ≥ 5 mm in diameter are considered to be infected) divided by the total number of assessed fruits × 100].

In September 2019, six kiwifruit plants were randomly selected from a planting of kiwifruit growing under OF or RS cultivation systems. A total of 40 mid-size healthy fruits were hand-harvested from each replicate kiwifruit plant and immediately placed in sterile bags that were placed on ice and transferred to the lab for further processing. Each group has three biological replicates. All personnel involved in sample collection wore facial masks and sterile gloves to ensure sterility. Different gloves were strictly used when taking different samples to avoid contamination.

Once in the laboratory, 40 kiwifruits in each replicate group were randomly divided into two groups for the assessment of epiphytes or endophytes. Three biological replicates were used for each of the sampled groups and each replicate comprised 40 fruits. For the collection of epiphytic microbes, sterilized phosphate buffered saline (PBS) solution was added to a sterile bag containing 20 kiwifruits until the fruits were completely covered. The bags were then placed on rotary shaker at 25°C and 100 rpm for 30 min. After shaking, the solution was filtered through a 0.22 μm filter and then the membrane was placed in a centrifuge tube, frozen in liquid nitrogen, and stored at −80°C until further processing. For the collection of endophytic microbes, kiwifruit peels were firstly removed using a sterilized knife. The remaining pulp from 20 kiwifruits were cut into small pieces and placed in a sterile bag, followed by the addition of enough PBS solution to generously cover the pieces of pulp. The bags were then placed on a rotary shaker at 25°C and 100 rpm for 30 min. After shaking, the solution was filtered with a sieve and then loaded into a centrifuge tube. After centrifugation at 4°C and 7000*g* for 30 min, the supernatant was removed, frozen in liquid nitrogen, and stored at −80°C until further processing.

### Library Preparation and Amplicon Sequencing

Total genomic DNA was extracted from each kiwifruit sample using the CTAB method with slight modification (Kim et al., 2019). The quality and quantity of the extracted DNA were evaluated using a NanoDrop spectrophotometer (Thermo Fisher Scientific, Cleveland, OH, United States). The extracted DNA was then eluted in 50 μL of Elution buffer and subsequently used as a template for PCR amplification. The V3-V4 variable regions of the 16S ribosomal RNA (rRNA) gene was amplified for assessing bacterial diversity using the primers 341F (5′-CCTACGGGNGGCWGCAG-3′) and 806R (5′-GGACTACHVGGGTWTCTAAT-3′). The ITS2 variable region of the small-subunit rRNA gene was amplified for assessing fungal-diversity using the primers ITS3-KYO2F (5′-GATGAACGYAGYRAA-3′) and ITS4-2409 (5′-TCCTCCGCTTATTGATATGC-3′). The PCR conditions consisted of an initial denaturation at 94°C for 2 min; followed by 30 cycles consisting of denaturation (98°C for 10 s), annealing at 62°C for 30 s, and extension at 68°C for 30 s; and then final extension at 68°C for 5 min. The expected size of the 16S and ITS2 amplified fragments generated by each pair of primers was 466 and 381 bp, respectively. The 5′ ends of the primers were also tagged with specific barcodes for each sample.

After amplicon generation, the PCR products were assessed using 2% agarose gel electrophoresis and further purified using AMPure XP beads (Beckman Coulter Genomics, Danvers, MA, United States). The obtained amplicons were quantified using a Qubit dsDNA Assay Kit (Invitrogen, Waltham, MA, United States). Equal amounts of purified amplicons from each sample were pooled into libraries for high-throughput sequencing. High-throughput amplicon sequencing assay was performed on an Illumina Novaseq 6000 platform according to standard protocols (Illumina, San Diego, CA, United States).

### Data Analysis

The raw, paired-end sequence data were first assigned to their respective samples based on their barcodes. Reads without barcode sequences were discarded. The remaining paired-end reads were merged using FLASH software with default parameters (Reyon et al., 2012). Further processing of the merged reads was done using CLC genomics workbench 20.^1^ Clean reads were obtained after trimming the barcodes and primer sequences, removing shorter reads (a minimum size of 300 and 200 bp for 16S and ITS2 amplified fragments, respectively), filtering low quality reads with a quality value (Q) less than 20, and cleaning up contaminated reads through alignment (*E*-value ≤ *e*-05 and identity ≥ 80%) against the kiwifruit genome^2^ (Yue et al., 2015). Chimeric sequences were also identified and filtered using VSEARCH software (version 2.3.4) to obtain high-quality clean reads (Rognes et al., 2016). The UCLUST algorithm in QIIME 1.9.1 software (Caporaso et al., 2010) was used to cluster the high-quality clean reads by alignment to the SILVA Database (version 132) (Pruesse et al., 2007) for 16S rRNA sequences and the UNITE dynamic database (Abarenkov et al., 2010) for ITS sequences. Sequences with ≥97% similarity were assigned to a specific operational taxonomic unit (OTU) and the most abundant sequence was selected as representative of each OTU (Edgar, 2013). The representative OTUs were annotated to varying taxonomic ranks, including phylum, class, order, family, genus, and species. The rarefied OTU data was used to calculate α and β diversity indices. For α diversity, the richness of each sample was evaluated using Chao1 metrics and the diversity within each sample was estimated using the observed richness (Shannon) index. For β diversity, PCoA analysis was conducted based on the Unweighted Unifrac distance, and the principal coordinate combination with the largest contribution rate was selected for plotting. PCoA analysis was conducted using the R project Vegan package (version 2.5.3) and used to compare bacterial and fungal community structure between samples. SPSS was used for significant analysis of the relative abundance of the samples between the groups. Independent sample *T* test was used for comparison between the two groups, and single-factor ANOVA was used for analysis of the samples of the three groups or more, and the significant judgment condition was *p* < 0.05.

## Results

### Disease Incidence

The disease incidence at harvest was assessed in the two cultivars growing under the two cultivation systems (OF and RS) in 2 different years (2018 and 2019). The predominant causal pathogens were *A. alternata* and *Nigrospora oryzae*. The incidence of fruit disease was significantly lower in both cultivars for both diseases in kiwifruit cultivated under the RS compared to the OF (*p* < 0.05). The disease incidence of the “Hongyang” and “Cuiyu” cultivars was 5.7 and 2.2%, respectively, under RS conditions in 2018, while it was 15.4 and 17.4% in the two cultivars, respectively, under OF conditions (Table 1A). Similarly, in 2019, the disease incidence of the “Hongyang” and “Cuiyu” cultivars was 5.3 and 3.3%, respectively, under RS conditions, and 12.3 and 15.6%, respectively, under OF conditions (Table 1B).

**TABLE 1:** Pre-harvest incidence of natural disease in “Hongyang” and “Cuiyu” kiwifruit cultivars in 2018 **(A)** and 2019 **(B)** grown under rain-shelter and open-field cultivation systems.

**Variety**	**Cultivation methods**	**Incidence rate %**
**A**		
Hongyang	rain-shelter	5.7 ± 0.4^b^
Hongyang	open-field	15.4 ± 1.2^a^
Cuiyu	rain-shelter	2.2 ± 0.3^b^
Cuiyu	open-field	17.4 ± 1.5^a^
**B**		
Hongyang	rain-shelter	5.3 ± 0.5^b^
Hongyang	open-field	12.3 ± 1.1^a^
Cuiyu	rain-shelter	3.3 ± 0.3^b^
Cuiyu	open-field	15.6 ± 1.2^a^

*No disease management practices were administered during the fruit production season. Significant differences (*p* < 0.05) between rain-shelter and open-field groups within the same cultivar (“Hongyang” or “Cuiyu”) are indicated by different letters.*

### High-Throughput Amplicon Sequencing

The high-throughput sequencing generated 5192782 16S rRNA and 5103950 fungal ITS raw sequences. A total of 5023302 and 5052962 bacterial and fungal clean reads were obtained, respectively, after the removal of chimeras and low-quality sequences. The resulting clean reads were classified into 3639 bacterial and 2863 fungal OTUs based on a 97% sequence similarity. The bacterial OTUs were assigned to 5 phyla, 16 orders, and 23 genera while the fungal OTUs were assigned to 2 phyla, 18 orders, and 19 genera.

### Composition Analysis of the Bacterial Community

**FIGURE 1:**
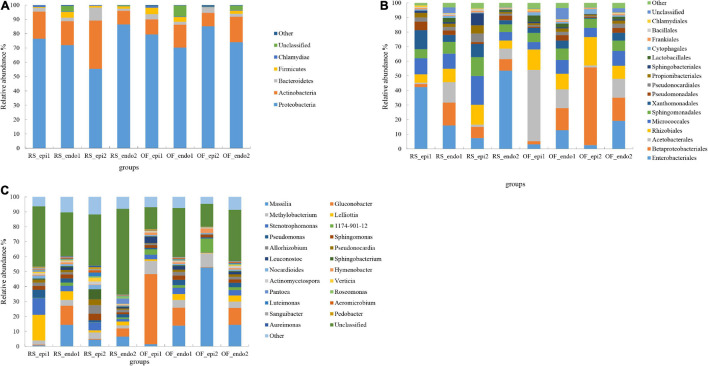
Relative abundance of bacterial taxa at the level of phylum **(A)**, order **(B)**, and genus **(C)**. Bacterial taxa with a relative abundance <1% were grouped together and are designated as “Other.” Samples labeled “RS” and “OF” designate rain-shelter (RS) and open-field (OF) samples, respectively. The terms “epi” and “endo” designate epiphytic and endophytic sample groups, respectively, and the designations “1” and “2” indicate “Hongyang” and “Cuiyu” cultivars, respectively. The samples were taken in September 2019.

The phyla, Proteobacteria, Actinobacteria, Bacteroidetes, Firmicutes, and Chlamydiae accounted for 75, 18, 3, 2, and 0.7% of the assigned OTUs, respectively (Figure 1A). The relative abundance of Proteobacteria in RS_epi1 (RainShelter-Epiphytic-“Hongyang”) samples was significantly higher (*p* < 0.05) than in RS_epi2 (RainShelter-Epiphytic-“Cuiyu”) samples, while the relative abundance of Proteobacteria in RS_endo1 (RainShelter-Endophytic-“Hongyang”) samples was significantly lower (*p* < 0.05) than it was in RS_endo2 (RainShelter-Endophytic-“Cuiyu”) samples. The proportion of Proteobacteria in OF_epi1 (OpenField-Epiphytic-“Hongyang”) samples was significantly (*p* < 0.05) lower than in OF_epi2 (OpenField-Epiphytic-“Cuiyu”) samples. The proportion of Actinobacteria in “Cuiyu” samples with RS cultivation was significantly higher (*p* < 0.05) than in “Hongyang” samples, with the order of abundance being RS_epi2 (33.79%) > RS_epi1 (19.01%). The proportion of Actinobacteria in RS samples was significantly higher (*p* < 0.05) than it was in OF samples, except for the relative abundance of OF_endo2 (17.76%), which was significantly higher (*p* < 0.05) than it was in RS_endo2 (9.56%). The highest relative abundance (9.02%) of Bacteroidetes was observed in RS_epi2 samples, followed by OF_epi2 (3.91%), OF_epi1 (3.61%), and RS_epi1 (2.57%). The highest proportion of Bacteroidetes in endophyte samples was observed in the RS_endo1 sample.

At the order level. Enterobacteriaceae was dominant in RS_epi1 samples, having a relative abundance of 42.31% (Figure 1B). The relative abundance of Enterobacteriaceae was greater (*p* < 0.05) in RS samples than in corresponding OF samples. In regards to epiphytes, the relative abundance of Enterobacteriaceae was significantly higher (*p* < 0.05) in “Hongyang” than in “Cuiyu,” while of the opposite was true for endophytic Enterobacteriaceae. Betaproteobacteriales, an order within the Proteobacteria, was the dominant order in OF_epi2 (53.24%) samples. The relative abundance of Betaproteobacteriales in epiphytic samples was higher in “Cuiyu” than in “Hongyang.” The relative abundance of Micrococcales in RS samples was significantly higher than (*p* < 0.05) it was in OF samples, with the exception that the proportion in RS_endo2 samples was significantly lower (*p* < 0.05) than it was in OF_endo2 samples. The relative abundance of Sphingomonadales in RS and OF samples was similar to the distribution observed for the Micrococcales. The percentage of Xanthonadales (13.05%) and Pseudomonadales (5.83%) in RS_epi1 samples was significantly higher (*p* < 0.05) than it was in the other sample groups, and the proportion of the two orders was significantly higher (*p* < 0.05) in “Hongyang” RS samples than it was in OF samples. The relative abundance of Propionibacteriales (5.61%), Pseudonocardiales (5.79%), and Sphingobacteria (8.30%) in RS_epi2 samples was significantly higher (*p* < 0.05) than in other sample groups.

Further analysis at taxonomic rank of genus revealed significant differences in the dominant genera present in the eight different sample groups, with the exception of the unclassified category (Figure 1C). *Massilia*, with a relative abundance of 52.62%, was the dominant genus in OF_epi2 samples. The relative abundance of epiphytic *Massilia* was significantly lower (*p* < 0.05) in RS samples than it was in OF samples. The relative abundance of *Methylobacterium* was significantly higher (*p* < 0.05) in “Cuiyu” epiphytic samples than in “Hongyang” epiphytic samples, while the opposite trend was observed in endophytic *Methylobacterium*. The proportion of *Methylobacterium* was significantly higher (*p* < 0.05) in OF samples than in RS samples. The relative abundance of epiphytic *Stenotrophomonas* within the same cultivar, was significantly higher (*p* < 0.05) in RS samples than in OF samples. The relative abundance of *Sphingomonas* was 2.47 and 4.38% in RS_epi1 and RS_epi2, respectively, both of which were significantly higher (*p* < 0.05) than they were in other OF groups. The highest proportion (5.47%) of *Pseudomonas*, among all samples, was observed in RS_epi1 samples, and the relative abundance of *Pseudomonas* was significantly higher (*p* < 0.05) in RS epiphytic samples than in OF epiphytic samples. The highest level of relative abundance (6.68%) of *Sphingobacterium* was observed in RS_epi2 samples while its abundance in other sample groups ranged from 0.06 to 0.79%.

### Composition Analysis of the Fungal Community

**FIGURE 2:**
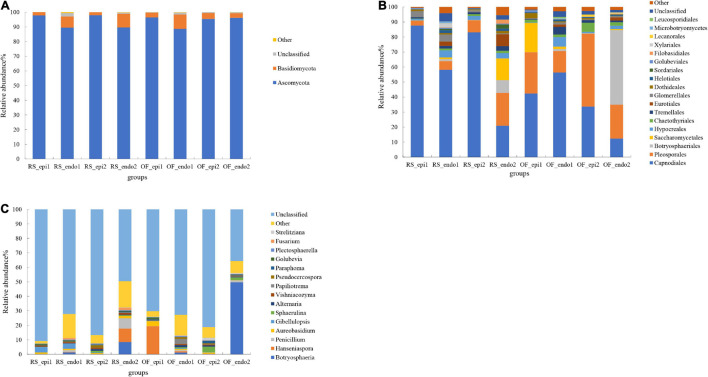
Relative abundance of fungal taxa at the level of phylum **(A)**, order **(B)**, and genus **(C)**. The fungal taxa with a relative abundance <1% were grouped together and are designated as “Other.” Samples labeled “RS” and “OF” designate rain-shelter (RS) and open-field (OF) samples, respectively. The terms “epi” and “endo” designate epiphytic and endophytic sample groups, respectively, and the designations “1” and “2” indicate “Hongyang” and “Cuiyu” cultivars, respectively. The samples were taken in September 2019.

Ascomycota and Basidiomycota were the dominant phyla in all sample groups. with Ascomycota being the most abundant phylum, accounting for 88.66–98% of the OTUs in all sample groups (Figure 2A). The proportion of Ascomycota was significantly higher in RS samples than in OF samples. Within the same cultivation system, the relative abundance of Ascomycota was significantly higher (*p* < 0.05) in “Cuiyu” than in “Hongyang” samples, except that the proportion in OF_epi1 (“Hongyang”) was higher than it was in OF_epi2 (“Cuiyu”). The relative abundance of Basidiomycota in the sample groups ranged from 1.91 to 9.7%. Notably, the abundance of epiphytic fungi within the same cultivar was significantly lower (*p* < 0.05) in RS samples than in OF samples.

The dominant orders of fungi were Capnodiales, Pleosporales, Botryosphaeriales, Saccharomyceteles, and Hypocreales (Figure 2B). The proportion of Capnodiales was higher in RS samples than it was in OF samples, and the relative abundance of epiphytic Capnodiales was significantly higher (*p* < 0.05) in RS_epi1 and RS_epi2 (87.63 and 83.03%, respectively) samples than it was in OF_epi1 and OF_epi2 (42.36 and 33.7%, respectively) samples. Pleosporales accounted for 48.47% of the OTUs in OF_epi2 samples and within the same cultivation system, the proportion of Pleosporales was significantly lower (*p* < 0.05) in “Hongyang” samples than in “Cuiyu” samples. Within the same cultivar, OF samples had a higher level of Pleosporales than RS samples. The relative abundance of Botryosphaeriales was the highest (49.2%) in OF_endo2 samples and the relative abundance of endophytic Botryosphaeriales was significantly higher (*p* < 0.05) than for the relative abundance of epiphytic. Saccharomycetales accounted for 19.43 and 14.3% of the identified OTUs in OF_epi1 and RS_endo2 samples, respectively. The relative abundance of Saccharomycetales was significantly lower (*p* < 0.05) in RS samples than in OF samples, except for RS_endo2. The relative abundance of epiphytic Hypocreales, within the same cultivation system, Hypocreales was significantly lower (*p* < 0.05) in “Hongyang” than it was in “Cuiyu,” while the opposite trend was observed for endophytic Hypocreales, the abundance of epiphytic Hypocreales was significantly higher (*p* < 0.05) in RS samples than in OF samples.

Analysis of the composition of the fungal community was also conducted at the genus level (Figure 2C). Results revealed that some genera of pathogenic fungi were present in kiwifruit peel and pulp tissues, such as *Botryosphaeria*. The relative abundance of *Botryosphaeria* in OF_endo2 was 49.62%, which was the same proportion observed at level of order. The relative abundance of *Penicillium* in RS_endo2 was 7.04%, which was significantly higher (*p* < 0.05) than its abundance as an epiphyte. The relative abundance of epiphytic *Penicillium*, within the same cultivation system, was significantly higher (*p* < 0.05) in “Hongyang” than it was in “Cuiyu.” Within the same cultivar, however, the level of epiphytic *Penicillium* was significantly lower (*p* < 0.05) in RS samples than in OF samples. The relative abundance of *Hansniapora* in OF_epi1 was 19.39%, which was significantly higher (*p* < 0.05) than it was in other groups. *Vishniacozyma* accounted for 0.19–1.1% of the OTUs in all samples, within the same cultivar, the proportion of *Vishniacozyma* was significantly higher (*p* < 0.05) in RS samples than it was in OF samples. The relative abundance of *Vishniacozyma*, within the same cultivation system, was significantly lower (*p* < 0.05) in “Hongyang” than it was in “Cuiyu.” The proportion of epiphytic *Plectosphaerella*, within the same cultivar, was significantly higher (*p* < 0.05) in RS samples than it was in OF samples.

### α Diversity Analysis of Bacterial and Fungal Communities

The Shannon index of the bacterial community in RS_epi2 samples was significantly higher (*p* < 0.05) than it was in other groups (Table 2), while the Shannon index of OF_epi1 and OF_epi1 were the lowest. No significant differences in diversity were observed between OF_endo1, RS_endo1, and OF_endo2 samples. The Chao1 index of the bacterial community in epiphytic samples was significantly higher (*p* < 0.05) than it was in endophytic groups.

**TABLE 2:** α diversity indices (Chao1 and Shannon) of the epiphytic and endophytic bacterial community of “Hongyang” and “Cuiyu” kiwifruit grown under rain-shelter and open-field cultivation systems.

**Groups**	**Chao1**	**Shannon**
RS_epi1	1146.7794.50^a^	5.25 ± 0.06^c^
RS_endo1	292.2247.50^bc^	5.75 ± 0.05^b^
RS_epi2	1158.7056.42^a^	6.15 ± 0.03^a^
RS_endo2	379.7646.33^b^	4.67 ± 0.31^d^
OF_epi1	1161.7629.43^a^	4.42 ± 0.07^d^
OF_endo1	267.7435.10^c^	5.67 ± 0.05^b^
OF_epi2	1147.3020.62^a^	4.64 ± 0.10^d^
OF_endo2	302.0327.50^bc^	5.68 ± 0.06^b^

*Significant differences (*p* < 0.05) between sample groups are indicated by different letters. Samples labeled “RS” and “OF” designate rain-shelter (RS) and open-field (OF) samples, respectively. The terms “epi” and “endo” designate epiphytic and endophytic sample groups, respectively, and the designations “1” and “2” indicate “Hongyang” and “Cuiyu” cultivars, respectively.*

The Chao1 index of the fungal community was highest in the OF_epi2 sample group, which contained the greatest number of species, followed by RS_epi2 and OF_epi1 (Table 3). The Chao1 index in epi2 sample groups, regardless of cultivation system, was higher than it was in epi1 samples. The highest Shannon index was observed in RS_endo2 and OF_endo1 sample groups, followed by RS_endo1 and OF_epi2, with RS_epi1 having the lowest Shannon index.

**TABLE 3:** α diversity indices (Chao1 and Shannon) of the epiphytic and endophytic fungal community of “Hongyang” and “Cuiyu” kiwifruit grown under rain-shelter and open-field cultivation systems.

**Groups**	**Chao1**	**Shannon**
RS_epi1	671.0260.93^c^	2.81 ± 0.02^e^
RS_endo1	249.6346.61^ef^	4.89 ± 0.06^ab^
RS_epi2	907.1561.91^b^	3.82 ± 0.04^c^
RS_endo2	183.0815.06^f^	5.08 ± 0.23^a^
OF_epi1	845.8955.66^b^	3.69 ± 0.04^cd^
OF_endo1	343.908.50^d^	5.03 ± 0.37^a^
OF_epi2	1026.042.05^a^	4.60 ± 0.04^b^
OF_endo2	274.2138.23^de^	3.46 ± 0.12^d^

*Significant differences (*p* < 0.05) between sample groups are indicated by different letters. Samples labeled “RS” and “OF” designate rain-shelter (RS) and open-field (OF) samples, respectively. The terms “epi” and “endo” designate epiphytic and endophytic sample groups, respectively, and the designations “1” and “2” indicate “Hongyang” and “Cuiyu” cultivars, respectively.*

### β Diversity Analysis of Bacterial and Fungal Communities

**FIGURE 3:**
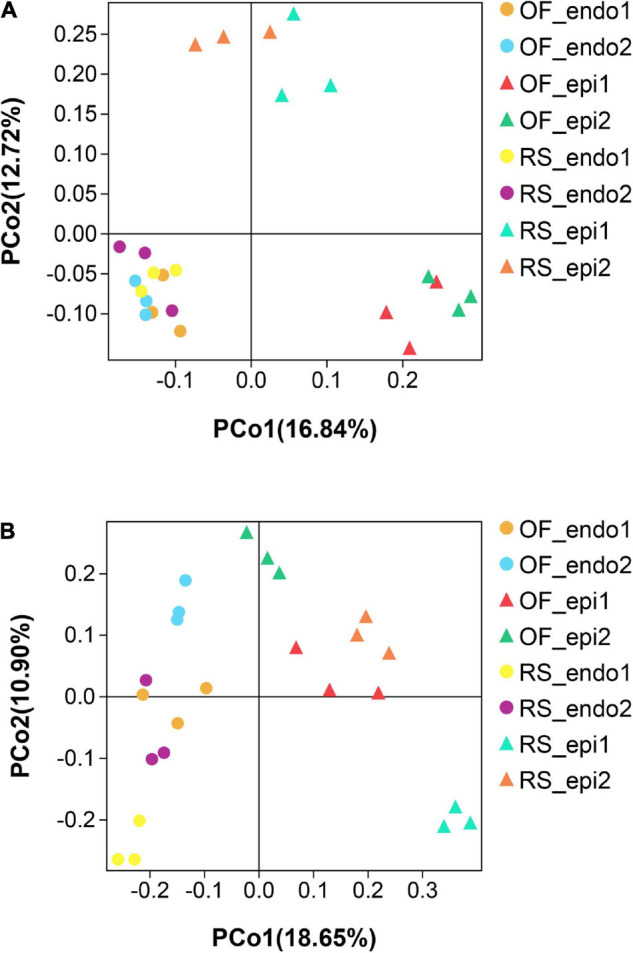
Principal Coordinate Analysis (PCoA) analysis of bacterial **(A)** and fungal **(B)** communities in the different kiwifruit sample groups based on unweighted_unifrac distance. Samples labeled “RS” and “OF” designate rain-shelter (RS) and open-field (OF) samples, respectively. The terms “epi” and “endo” designate epiphytic and endophytic sample groups, respectively, and the designations “1” and “2” indicate “Hongyang” and “Cuiyu” cultivars, respectively. In the figure, to facilitate the distinction, we use circles to represent endophytes and upward triangles to represent epiphytes. The samples were taken in September 2019.

**FIGURE 4:**
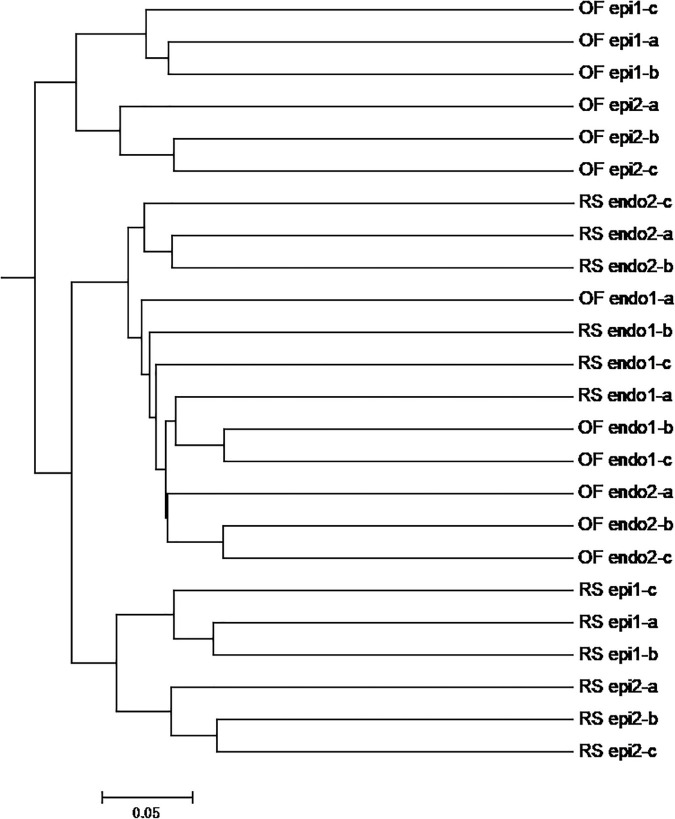
UPGMA analysis of OTUs of bacteria community in different kiwifruit samples based on unweighted_unifrac distance. Note: In the samples, the prefix “RS” and “OF” mean rain-shelter cultivation and open-field cultivation, respectively; “epi” and “endo” represent epiphytic and endophytic, respectively; “1” and “2” indicate “Hongyang” and “Cuiyu” cultivars, respectively.

The PCoA of the bacterial community (Figure 3A) exhibited three distinct clusters of samples. PCoA 1 accounts for 16.84% of the variation with the bacterial communities of the 24 sample groups placed in three clusters. One cluster is primarily composed of endophytic samples, while the other two clusters are mainly composed of epiphytic samples, with samples from the same cultivation system clustering near each other. PCoA 2 explains 12.72% of the variation. In PCoA 2 the epiphytic samples of the bacterial communities representing RS samples are located higher than the OF epiphytic samples. At the same time, the placement of RS_epi2 and RS_epi1 communities is closer, indicating that their species structure is more similar. RS_endo2 and RS_endo1 communities are at the same height in the vertical coordinates. Results of the PCoA analysis are also supported by an UPGMA clustering tree (Figure 4).

**FIGURE 5:**
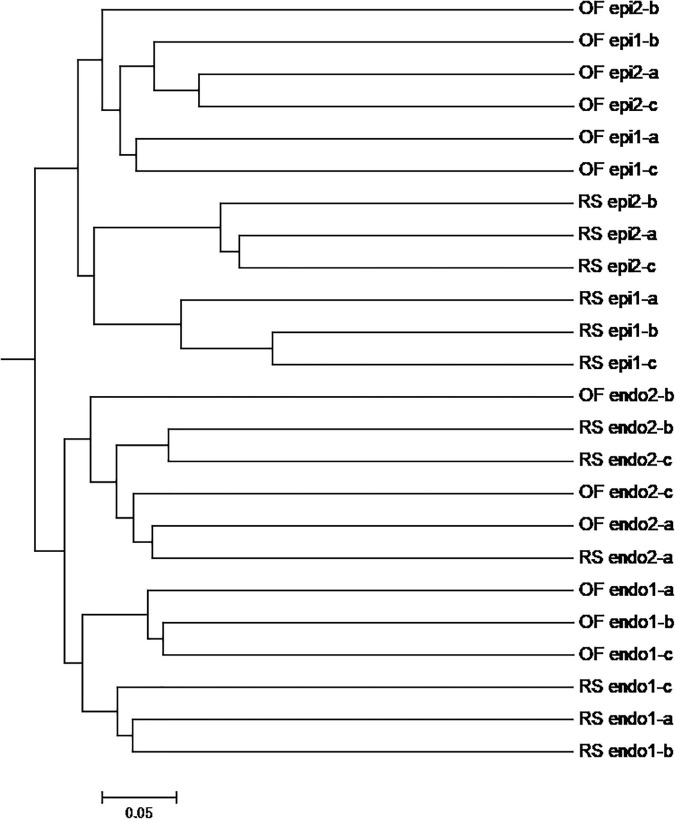
UPGMA analysis of OTUs of fungal community in different kiwifruit samples based on unweighted_unifrac distance. Note: In the samples, the prefix “RS” and “OF” mean rain-shelter cultivation and open-field cultivation, respectively; “epi” and “endo” represent epiphytic and endophytic, respectively; “1” and “2” indicate “Hongyang” and “Cuiyu” cultivars, respectively.

In the PCoA plot of the fungal community (Figure 3B), PCoA 1 explains 18.65% of the variation and the distance between epiphytic and endophytic communities is large and the distance between the fungal communities in the two different cultivations systems (RS and OF) is much closer. PCoA 2 explains 10.90% of the variation and the distance between the epiphytic OF samples is greater than the distance between the epiphytic RS samples. Among the endophytic samples, the distance between the fungal communities in “Cuiyu” (endo2) and “Hongyang” (endo1) was great. The results of the PCoA analysis were also supported by an UPGMA clustering tree (Figure 5).

## Discussion

Pre- and post-harvest diseases of fruits resulting from microbial pathogens result in significant economic losses. Similar to reports in other fruit crops (Meng et al., 2012; Claire et al., 2018), cultivation of kiwifruit under a RS system effectively reduced the level of plant disease, relative to OF cultivation. While air pollutants are spread by wind, they are often deposited on plants and the soil surface by rainfall, which may directly or indirectly increase the incidence of both foliar and soilborne diseases (Dimabuyu et al., 2016). Compared with the OF cultivation, The humidity level and soil organic matter content are often higher in RS systems than OF systems, which can provide a more stable growth environment for plant growth (Lim et al., 2016).

The data in Table 1 indicate that disease incidence was significantly reduced (*p* < 0.05) when kiwifruit was grown under a RS. The level of disease incidence, however, differed between the two varieties of kiwifruit. The level of disease incidence was higher in “Hongyang” kiwifruit than it was in “Cuiyu.” “Hongyang” kiwifruit has been reported to be more susceptible to canker and *Penicillium* than other kiwifruit varieties (Luo et al., 2019; Zhao Z. et al., 2019). The plant host and its resident microbiota interact with each other. In the present study, we characterized the epiphytic and endophytic community composition in the two different cultivars grown under two different cultivation systems (OF and RS) to determine if there was an association between the composition of the microbial community and the observed levels of disease incidence.

The phyla, Proteobacteria, Actinobacteria, Bacteroidetes, and Firmicutes have been reported to be widely distributed on the fruit surface of plant species, and contain a variety of taxa with different ecological roles, functioning as antagonists, symbionts (especially endophytes), and saprophytes (Insuk et al., 2020; Lee et al., 2020; Mamphogoro et al., 2020). Actinobacteria have been reported to have a higher abundance in the endophytic community of kiwifruit cultivars that are more resistant to bacterial canker (Cho et al., 2018). Insuk et al. (2020) assessed the *in vitro* and *in vivo* growth- promoting activity of 13 strains of Actinomycetes isolated from bryophytes. They found that all of the tested isolates produced IAA and also sequestered iron, which promoted the growth of bryophytes. In the present study, Actinobacteria, exhibited a higher abundance in RS samples than in OF samples, within the same cultivar (Figure 1A). The genera *Pseudomonas*, *Sphingomonas*, and *Methylobacterium* have also been reported to be antagonists of fungal pathogens, and to reduce the biomass of *Fusarium oxysporum* and other fungal pathogens by inhibiting their growth, thus, ameliorating disease symptoms (Mamphogoro et al., 2020). The proportion of *Sphingomonas* observed in our study was higher in RS samples than in OF samples and the proportion of *Methylobacterium* was higher in “Cuiyu” epiphytic samples than it was in epiphytic samples of “Hongyang” (Figure 1C). Some strains of *Sphingomonas* have been reported to induce resistance to *Fusarium graminearum* in host plants (Innerebner et al., 2011; Cho et al., 2018). Moreover, the number of colonies of *P. syringae* obtained from tomato leaves and the population of *Xanthomonas campestris* in leaves of *Arabidopsis* were significantly reduced, relative to a control, when plants were inoculated with *Sphingomonas* (Purahong et al., 2018). Notably, healthy kiwifruits were found to contain a greater diversity and abundance of bacterial taxa belonging to *Methylobacterium*, *Sphingomonas*, and *Nocardioides* than diseased fruit (Wu et al., 2019). Some species of *Pseudomonas* synthesize antibacterial metabolites, such as 2,4-diacetyl phloroglucinol and hydrogen cyanide. These two compounds have been reported to be the main active compounds responsible for the biocontrol activity of *Pseudomonas* in different agricultural ecosystems (López et al., 2020). Therefore, it is plausible to suggest that these beneficial bacteria may play a role in reducing the level of disease incidence observed in kiwifruit grown under a RS system.

Surveys have indicated that Ascomycota is the most abundant fungal phylum in soils throughout the world (Yousef et al., 2018). Different species of Ascomycota have been used to improve the quality of food crops and several species exhibit a strong tolerance to heavy metals (Lin et al., 2019). The growth rate of species of Ascomycota in soils is linked to soil nitrogen availability, and Ascomycetes play a major role as decomposers in soils (Liu et al., 2020). The order Capnodiales, within the Ascomycota, is common on the surface of leaves, and the abundance of this taxa is dependent on the level of soil organic matter (Detheridge et al., 2020). The relative abundance of Ascomycota and Capnodiales in our study was higher in RS samples than it was in OF samples (Figures 2A,B), suggesting that the soil environment under the RS may have had a higher content of organic matter, making it more suitable environment for Capnodiales and for plant growth. The higher organic matter content in the RS system can be attributed to its relatively closed environment, such as occurs in a greenhouse, where soil microorganisms may be the main source of epiphytic fruit fungi (Dong et al., 2021). Species in the order Hypocreales have been reported to exhibit an inhibitory effect on mycelial growth, hyphal formation, and ascospore germination, of the fungal pathogen, *Sclerotinia sclerotiorum*, on soybean plants (Peitl et al., 2020). Therefore, members of this order are ideal candidates for use as biological control agents. Our results indicated that the abundance of Hypocreales was lower in “Hongyang” samples than in “Cuiyu” samples (Figure 2B), indicating that there were a greater number of beneficial fungi present in “Cuiyu” than in “Hongyang” fruit, which was also reflected in the lower disease incidence observed in RS samples of “Cuiyu.”

Soft rot is responsible for severe economic losses in many fruits. The order Botryosphaeriales contains several species causing soft rot (Li et al., 2017). The relative abundance of Botryosphaeriales within the same cultivation system (RS vs. OF), and within the same cultivar, was significantly lower (*p* < 0.05) in RS samples than it was in OF samples (Figure 2B). The relative abundance of *Botryosphaeria* as endophyte was relatively high compared to its abundance as an epiphyte (Figure 2C), suggesting that it may represent a latent pathogen in kiwifruit. In this regard, *B. dothidea* has been shown to colonize healthy tissues of woody plants as an endophyte where it remains latent until specific environmental cues trigger the onset of infection and disease, although the specific cues and infection mechanism of *B. dothidea* remain to be elucidated (Kim et al., 1999). The genus Hanseniaspora, displayed in Figure 2C, has been used as a biocontrol agent. For example, Ruiz-Moyano et al. (2020) reported that *H. uvarum* strain793, can be used to decrease the incidence of gray mold (*B. cinerea*) in strawberries and cherries. Lutz et al. (2020) combined *Vishniacozyma victoria* NPCC 1263 with CaCl_2_, and found that the combination effectively inhibited *Penicillium expansum* and *B. cinerea* on pear fruit, and that *V. victoria* readily colonized the fruit surface. Kusstatscher et al. (2019) reported that microbial diversity was significantly lower (*p* < 0.05) in rotten beets (*Beta vulgaris*), relative to non-infected beets. In that study, *Plectosphaerella* and *Vishniacozyma* were the dominant genera present on healthy sugar beets. Notably, in our study, the proportion of these two genera was higher in RS samples than in OF samples.

All fruits and vegetables possess a rich, post-harvest microbiota that is specific to the host species and includes both beneficial and pathogenic microorganisms. Microbial diversity and microbial networks have been shown to be linked to the health of fruits and vegetables, with diseased products exhibiting a significant microbial imbalance (Kusstatscher et al., 2020). Purahong et al. (2018) compared differences in the bacterial community of healthy and infected tissues of two different kiwifruit varieties. They found that the population evenness and biodiversity of “Hongyang” (flowers and leaves) and “Hayward” (leaves only) infected with *P. syringae* pv. actinidiae was significantly lower (*p* < 0.05) than they were in healthy fruit and that infected tissues possessed only a relatively low number of genera, with *Pseudomonas* being the most dominant. Williams and Marco (2014) found that the number of bacterial species on lab-grown lettuce was much lower than the number of species on field-grown lettuce. The potential sources of bacteria in the field include seeds, irrigation water, soil, human contact, dust, and air. That study may help to explain the differences observed in the Shannon index for epiphytic bacteria between RS and OF samples of kiwifruit (Table 2). The Shannon index of epiphytic RS samples was significantly higher (*p* < 0.05) than it was for epiphytic OF samples of kiwifruit within the same cultivar (Table 3). Zhang et al. (2020) reported that RS cultivation significantly increased the Shannon index of the rhizosphere bacterial community in pear and suggested that pear quality could be improved by regulating the diversity of the soil microbial community.

We characterized differences in the composition and structure of the bacterial and fungal communities in different sample groups of kiwifruits at the level of phylum, order, and genus. Our results indicated that the distribution of Enterobacteriales and Betaproteobacteriales varied in endophytic and epiphytic bacterial communities, and that the abundance of *Methylobacterium* and *Stenotrophomonas* was much lower in endophytic bacterial samples than it was in epiphytic bacterial samples. These latter results may explain the observed distance between epiphytic and endophytic bacterial communities in the PCoA plot (Figure 3A). Differences between endophytic bacterial samples, however, are not as obvious as they are for epiphytic samples. Cui et al. (2020) reported that the use of reclaimed irrigation water on pepper (*Capsicum annuum*) plants had no effect on the endophytic bacterial community structure in roots and fruits, indicating that the endophytic bacterial community has a certain level of resiliency. In the current study, we found that Proteobacteria in OF_epi2 samples was significantly higher (*p* < 0.05) than it was in other groups, which again may help to explain why this group was more distant from other groups in the PCoA plot. The relative abundance of Actinobacteria, Bacteroidetes, Enterobacteriales, and Acetobacterales in RS sample groups was higher than in OF sample groups. *Sphingomonas*, *Pseudomonas*, and *Sphingobacterium* have been reported to have a positive impact on plant health and have been used as plant-growth-promoting bacteria (Innerebner et al., 2011; Cho et al., 2018). In our results, their relative abundance in RS samples was higher than it was in OF samples. Different cultivation systems appear to be able to alter the microbial community of fruits. Notably, the Shannon index of the RS_epi2 sample group was significantly higher (*p* < 0.05) than it was in other groups. Disease incidence was also the lowest in RS samples. Interestingly, microbial diversity was also reported to be higher in disease-resistant cultivars than susceptible cultivars. Zhang et al. (2020) reported that the Shannon and Chao1 indices of the bacterial community in kiwifruit grown in RS systems were significantly higher (*p* < 0.05) than kiwifruit grown in OF systems. In that study, the abundance of Proteobacteria was significantly lower (*p* < 0.05) in RS samples than it was in OF samples. Similar to our results, the level of total nitrogen and total potassium were also significantly higher (*p* < 0.05) in RS soils than in OF soils.

In the fungal community, Botryosphaeriales, *Penicillium*, and Hypocreales accounted for a greater percentage of the endophytic community than they did in the epiphytic community in all samples. Differences in the relative abundance of these fungal genera had a distinct impact on the differences in fungal community composition observed between epiphytic and endophytic samples. The proportion of Ascomycota, including Capnodiales, *Vishniacozyma*, and *Plectosphaerella*, was higher in RS samples than in OF samples Within the same cultivar of kiwifruit. Basidiomycota and Saccharomycetales, however, accounted for a higher proportion of the taxa in OF samples. The proportion of Pleosporales in OF_epi2 was close to 50%, which may explain the separation of these samples from other epiphytic samples in the PCoA plot (Figure 3B). Wassermann et al. (2019) compared microbial community abundance in healthy and rotten apples and found that the diversity of bacteria and fungi in rotten apples was significantly lower (*p* < 0.05). The characteristics of a “healthy” apple microbial community were high diversity and evenness of bacterial and fungal taxa, while the microbial community in diseased apples exhibited a biological “imbalance” characterized by a loss of diversity, with pathogenic taxa becoming the dominant fungi. A greater abundance of bacterial genera, such as *Sphingomonas*, *Pseudomonas*, and *Methylobacterium* were found in healthy apples, as well as the fungal genera, *Vishniacozyma*, *Cladosporium*, and *Acremonium*. These findings suggest that regulating the composition of the fruit microbiota may represent a promising strategy for preventing post-harvest decay.

The comprehensive analysis of species composition, α diversity, and β diversity conducted in the present study, revealed differences in the number and abundance of species in the microbial community of kiwifruit between the fungal disease-resistant cultivar, “Cuiyu,” and the susceptible cultivar, “Hongyang.” Some distance in the clustering of the bacterial and fungal communities of RS and OF samples was also observed, indicating that RS cultivation can improve species richness. We found that Actinobacteria, Bacteroidetes, Enterobacteriales, Acetobacterales, *Sphingomonas*, *Pseudomonas*, and *Sphingobacterium* accounted for a large proportion of the bacterial community of “Cuiyu” kiwifruit cultivated under a RS, and that the fungal taxa, Capnodiales, Hypocreales, *Vishniacozyma*, and *Plectosphaerella* exhibited a high relative abundance. Similar patterns in community composition, genera, and relative abundance have been reported to exist in healthy plant tissues (Wu et al., 2019). Therefore, we suggest that the differences observed in the microbial community of kiwifruit between cultivars and between cultivation systems may contribute to the lower incidence of natural disease in kiwifruit observed under RS conditions and within the Cuiyu cultivar. Yu et al. (2020) applied a combination of three species of *Bacillus* on blueberries and found that the inoculated plants had a 14.56% increase in yield, relative to the untreated control, and that application of the bacterial consortium improved the quality of blueberry fruits. Beneficial bacteria can also increase the level of available nitrogen and organic matter in soils and are an important component of sustainable agricultural systems. The present study provides evidence that regulating the composition of the microbial community through cultivar selection and cultivation system can increase the abundance of beneficial bacterial taxa, resulting in reduced levels of disease (Daranas et al., 2018; Wang et al., 2019). Future research should focus on identifying the most effective species and strains of bacteria and/or fungi for fostering a beneficial microbial community, and more research should be conducted on the interactions that occur between members of the microbial community that may be responsible for the enhanced and synergistic impact of the microbial community on plant health and productivity (Tontou et al., 2016; Kusstatscher et al., 2020).

## Conclusion

A 2-year study indicated that RS cultivation reduces the level of disease incidence in kiwifruit. Further analysis of the microbial community of kiwifruit demonstrated that RS cultivation can affect the composition of the microbial community of kiwifruit. RS samples exhibited a greater abundance of beneficial microbial taxa, as well as a higher epiphytic microbial diversity, relative to samples obtained from OF plants. The composition and relative abundance of the fruit microbial community may potentially be used to evaluate the health status of fruits before and after harvest. A future goal is to effectively utilize a beneficial microbial community in conjunction with the use of biocontrol agents in support of ecological approaches for the production of kiwifruit.

## Data Availability Statement

The data sets generated for this study can be found at NCBI Accession No. PRJNA669580.

## Author Contributions

QY, HZ, and JiaL: conceptualization. QW, QY, and HZ: project administration. JianL, JinL, and QL: resources. QZ, ZW, and JY: data curation. YS and HZ: writing—original draft. JiaL, MJ, ZW, QY, and HZ: writing—review and editing. All authors contributed to the article and approved the submitted version.

## Conflict of Interest

The authors declare that the research was conducted in the absence of any commercial or financial relationships that could be construed as a potential conflict of interest.

## Publisher’s Note

All claims expressed in this article are solely those of the authors and do not necessarily represent those of their affiliated organizations, or those of the publisher, the editors and the reviewers. Any product that may be evaluated in this article, or claim that may be made by its manufacturer, is not guaranteed or endorsed by the publisher.

## Footnotes

1
https://www.qiagenbioinformatics.com/

2
http://kir.atcgn.com/
